# Regrouping induces anhedonia-like responses in dairy heifers

**DOI:** 10.3168/jdsc.2020-0023

**Published:** 2020-10-29

**Authors:** Benjamin Lecorps, Daniel M. Weary, Marina A.G. von Keyserlingk

**Affiliations:** Animal Welfare Program, Faculty of Land and Food Systems, 2357 Main Mall, University of British Columbia, Vancouver BC V6T 1Z6

## Abstract

Intensively housed dairy cattle are commonly regrouped (mixed into a new social group) as part of routine farm procedures. This stressful procedure triggers heightened levels of agonistic behaviors and disrupts animals' time budgets. However, little is known regarding the effects of regrouping on cattle's affective states. The aim of this study was to explore whether regrouping (involving a change in both the social and physical environment) triggers anhedonia (i.e., the reduced ability to experience pleasure) in 6-mo-old dairy heifers, a phenomenon associated with negative mood. In this study, we assessed anhedonia using changes in the use of a mechanical brush. Holstein heifers (n = 16) were trained to use a mechanical brush and then given the opportunity to individually brush for 10 min every 2 d. Time spent brushing (during a 10-min brush test) was collected before, during, and after regrouping (2-d interval) with the assumption that heifers would reduce their use of the brush during regrouping. Each heifer was individually regrouped into a new social group composed of 12 older and unfamiliar heifers and allowed access to the brush at 8 and 56 h after the onset of regrouping. Immediately after the last test, each heifer was brought back to her original pen and allowed to mingle with familiar pen-mates before being tested again 2 and 4 d later. When tested 8 h after regrouping, heifers reduced time spent brushing by 44 ± 27% (95% confidence interval: −96.18 to −41.8) compared with before regrouping; however, no differences were detected 56 h after regrouping. There was no relationship between the intensity of the decrease in brush use and any behaviors (number of agonistic interactions received, time heifers spent resting, or synchronization at the feed bunk) recorded for the 8 h immediately before testing (i.e., between 0 and 8 h and between 48 and 56 h after regrouping). These results indicate that regrouping induces anhedonia-like responses in dairy heifers on the day of regrouping. This routine procedure may thus induce negative mood in dairy heifers. This response was not related to behaviors typically collected to assess the negative effects of regrouping. Maintaining dairy cattle in stable social groups should be favored.

## Highlights

• Regrouping is a stressful experience for cattle
• Reduced ability to experience pleasure (anhedonia) indicates negative mood state
• We measured changes in brush use to assess anhedonia in 6-mo-old heifers
• Regrouping resulted in reduced brush use, indicative of low mood

## Graphical Abstract

**Figure:**
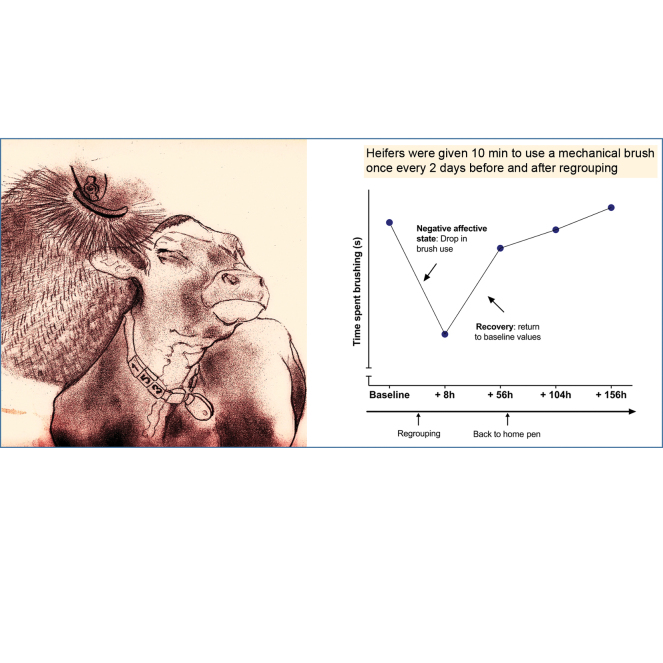
Summary: Regrouping (i.e., mixing individuals to form a new social group) is common on dairy farms. The practice is known to be stressful, but how cattle react emotionally to this stressor is still poorly understood. We studied whether heifers experience anhedonia (i.e., the reduced ability to experience pleasure) following regrouping by comparing their use of a mechanical brush before and after regrouping. Heifers reduced their use of the brush 8 h after regrouping, suggesting that they were experiencing anhedonia. This method shows promise for assessing affective states when animals are subjected to routine stressors.

Given that cattle are gregarious and motivated for social contact (Holm et al., 2002), group housing is assumed to provide benefits (Rault, 2012). However, in some circumstances, the social environment can be the source of acute or chronic social stress (Beery and Kaufer, 2015). Some routine practices such as social mixing (also termed “regrouping” or “commingling”) may have negative effects lasting for hours to days (Arey and Edwards, 1998; Patt et al., 2012). When regrouped, dairy cattle typically engage in more aggressive behaviors directed toward new members of the group (von Keyserlingk et al., 2008).

Although regrouping has been shown to cause negative physiological (Veissier et al., 2001) and behavioral effects in dairy cattle (von Keyserlingk et al., 2008; Nogues et al., 2020), little is known regarding the effects of this routine practice on the affective states of cattle. Stressors originating from the social environment may trigger negative affective states (Beery and Kaufer, 2015) and when applied as chronic stressors can induce depressive-like states in laboratory animals (Wang et al., 2017).

New methodologies have been developed to assess affective states and mood changes in dairy cattle (Ede et al., 2019), including anhedonia testing (Lecorps et al., 2019b). Anhedonia is defined as motivational and consummatory deficits toward pleasurable experiences (Treadway and Zald, 2011) and is typically associated with negative mood in humans and nonhuman animals (Rygula et al., 2005; Figueroa et al., 2015; Scheggi et al., 2018). In this study, our aim was to explore whether 6-mo-old juvenile dairy heifers display anhedonia in the hours and days after regrouping.

Cattle are motivated to use grooming devices (mechanical brushes; McConnachie et al., 2018), suggesting that access is rewarding. Thus, we first explored whether heifers' motivation to use a mechanical brush would be reduced after regrouping with unfamiliar conspecifics in an unfamiliar environment. We predicted that heifers would experience anhedonia on the day of regrouping but would return to baseline values on the days following, when behavioral changes associated with regrouping typically wane (von Keyserlingk et al., 2008). A second objective was to explore whether the individual change in brush use was related to animals' experiences during regrouping. Considering the negative effects of social defeat, we predicted that heifers subjected to more agonistic interactions would suffer from greater anhedonia. In addition, we expected that hunger and behavioral fatigue would have negative effects on heifers, predicting that animals that were less synchronized during feeding (feeding when feed was low in quality and in quantity) and that rested for shorter durations would show higher changes in brush use (i.e., higher anhedonia).

The study was approved by the University of British Columbia (UBC) Animal Care Committee (#A19-0128). We enrolled 16 Holstein heifers in this study (mean ± SD: 183.2 ± 19.2 d of age at the time of regrouping) that had been bred and raised at the UBC Dairy Education and Research Center. Before regrouping, heifers were housed in 2 stable groups of 8 animals in a pen consisting of a sawdust-bedded open pack (∼56 m^2^), fitted with a feed barrier with 13 feeding spaces. The regrouping pens consisted of 2 sand-bedded freestall pens (∼65 m^2^) equipped with 13 stalls (1.44 m^2^ per stall) and 16 headlocks. Heifers were fed a TMR and provided water ad libitum.

Every week, 2 heifers were regrouped (1 in each of 2 host groups) for 56 h before being brought back to their initial group. Once all heifers in the first group had been subjected to regrouping, a second group of heifers entered the study and the experiment was replicated. Regrouping involved a change in both the social and the physical environment; changes in the social environment involved regrouping with 12 unfamiliar heifers (mean ± SD) 259.3 d ± 28.4 d old, and changes in the physical environment involved moving from a bedded pack to freestalls and a change in the type of feed barrier. The host groups had been formed at least 2 wk before the first regrouping event. Regrouping occurred at 0800 h before feed delivery.

We first tested the effects of regrouping on brush use. Heifers were individually tested for anhedonia using a mechanical brush (mini swinging brush MSB, DeLaval, Tumba, Sweden). Briefly, animals were first habituated to the testing arena (sawdust-bedded open pack identical to the home pen described above) as a group (i.e., 3 sessions of 1 h/d per group). Then, heifers were brought in pairs to the testing arena every 2 d for 10 min until each heifer used the brush for more than 10 s in each of 2 consecutive sessions. The animals were then brought individually to the testing arena every 2 d for 10 min and the total time spent brushing was collected. Brush tests were always done at approximately 1600 h.

Baseline measures were taken 6, 4, and 2 d before regrouping and collected for at least 2 wk after heifers were first offered individual access to the brush. Animals were tested 8 and 56 h after regrouping. After the second test, heifers were brought back to their home pen and tested again 2 and 4 d later (Figure 1).

**Figure 1:**
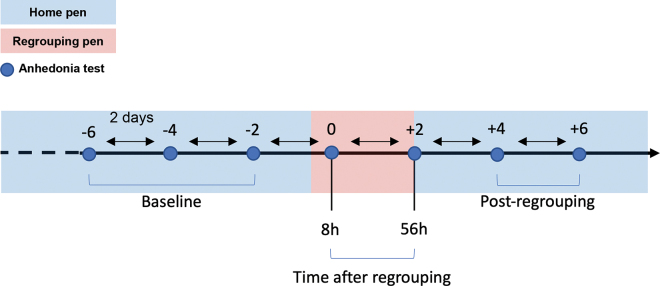
Timeline of the experimental procedure. Heifers (n = 16) were individually tested on the brush test before (3 times), during (2 times), and after regrouping (2 times). Tests were always separated by 48 h and took place at 1600 h. Regrouping took place at 0800 h with the first and second tests taking place 8 and 56 h later, respectively. Baseline was calculated using the average brush use of the 3 pre-regrouping tests. Brush tests always occurred in the same arena and away from the other pens.

Each focal heifer was tested individually and thus considered the statistical unit. Power analyses were run using the “pwr” function in R (https://www.R-project.org/); other statistical analyses were performed in SAS (version 9.4; SAS Institute Inc., Cary, NC). A sample size of 15 individuals was recommended for power set at 0.8, significance level set at 0.05, and a Cohen's d equal to 0.8. Of the 16 heifers tested, one was excluded because she failed to use the brush during the 3 baseline tests and another was injured during regrouping. The heifer was given medication and immediately returned to her initial group.

We first explored whether heifers used the brush less (compared with baseline) 8 and 56 h after regrouping using paired 2-sided *t*-tests. Data were checked for normality of the differences. Heifers used the brush on average (mean ± SD) 194.8 ± 110 s on baseline days. Animals reduced their use of the brush by (mean ± SD change) 43.5 ± 26.7% (Figure 2) 8 h after regrouping was initiated (*t*_14_ = 5.44, 95% CI = −96.18 to −41.8; *P* < 0.001). No change (with respect to baseline) was observed 56 h after regrouping (*P* > 0.05), indicating that overall animals returned to pre-regrouping values.

**Figure 2:**
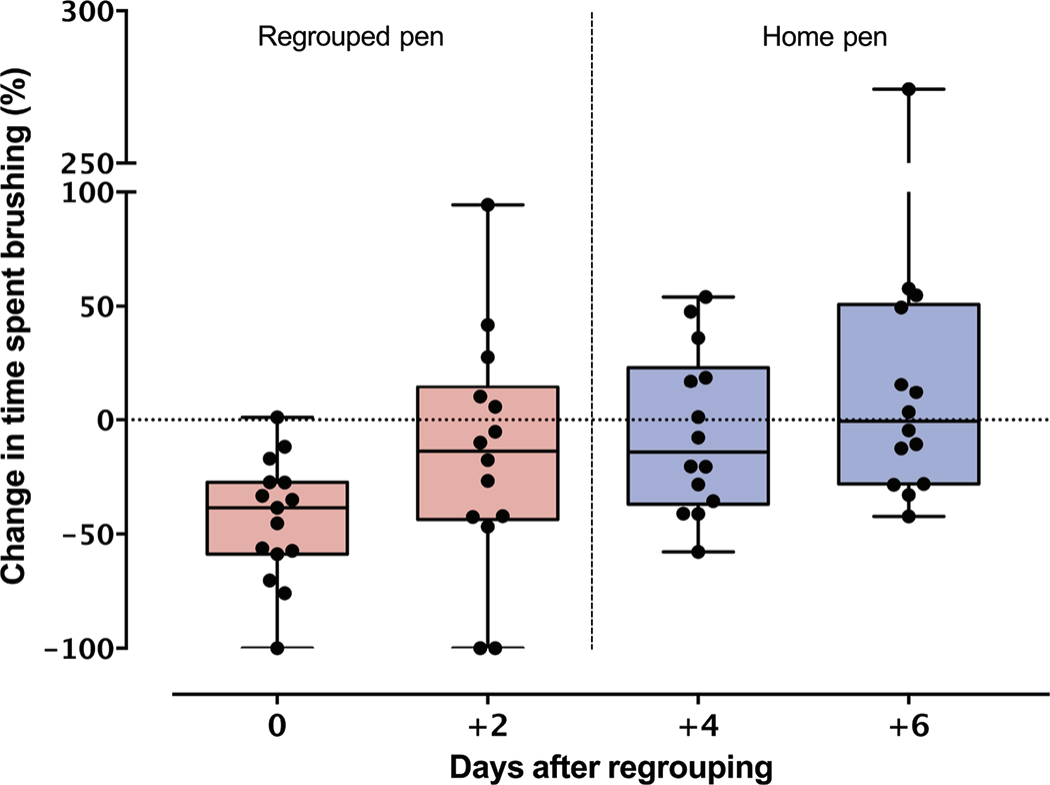
Change in brush use after regrouping and return to the home pen. Heifers (n = 15) were individually tested on the brush test before (3 times), during (2 times), and after regrouping (2 times). Baseline was calculated using the 3 pre-regrouping tests when animals were housed in their home pen. Percentage change was calculated for each of the post-regrouping time points. The test on d 0 was performed 8 h after regrouping and the test on d 2 was performed 56 h after regrouping. Tests on d 4 and 6 were performed 48 and 96 h after the return to the home pen, respectively. Tests were always performed 2 d apart at 1600 h. Brush tests always occurred in the same dedicated arena, away from the other pens. Boxes represent the interquartile ranges with median change; each dot is an individual point.

These results indicated that heifers showed evidence of anhedonia 8 h after regrouping but the effect waned over time (no effect 56 h after regrouping), a result consistent with previous results obtained by our group showing reduced interest in pleasurable resources such as milk after a stressful experience (i.e., hot-iron disbudding; Lecorps et al., 2019b). Hence, this study confirmed the utility of anhedonia testing to explore long-lasting negative affective states originating from routine farm procedures in dairy cattle (Ede et al., 2019). The use of a mechanical brush as a pleasant experience that is modulated by current mood states of dairy cattle is particularly promising and appears to be a suitable alternative to the sucrose preference test widely used in other species (Scheggi et al., 2018), but that may not be biologically relevant for weaned cattle. However, this new methodology will need further validation. Although the motivational aspect of brushing has been clearly established (i.e., cows were found equally motivated to access the brush as they were for fresh feed; McConnachie et al., 2018), brushing could also be seen as a coping mechanism after stressful experiences, as has been argued by some in the case of laboratory rodents (i.e., grooming patterns and frequency change in response to stressors; van Erp et al., 1994). Pharmacological or other experimental manipulations aimed to mitigate the negative affective states may be especially useful to confirm the sensitivity of the test to detect long-lasting negative affective states.

Our results also confirm that regrouping is a stressful experience that may trigger changes in mood in dairy heifers. Social defeats induced via chronic exposure to the resident-intruder test (where rats experience social defeats when introduced to a new and occupied environment; Rygula et al., 2005) or via weeks of various social stressors (e.g., unpredictable phases of isolation and crowding; Herzog et al., 2009) were found to trigger an anhedonic-like response. However, in our study, the stress-induced anhedonia was no longer detectable 56 h after regrouping. This result is consistent with previous studies showing that the negative effects associated with regrouping wane with time; neither agonistic interactions nor milk production differed from pre-regrouping values 3 d after regrouping in adult dairy cows (von Keyserlingk et al., 2008). However, our results also indicate that some animals maintained a low level of brush use when tested 56 h after regrouping, suggesting that some animals may still experience negative mood at this time. These animals may have taken longer to habituate to their new social environment because of personality differences or because their experience was more negative (e.g., they were subjected to more negative interactions).

We then examined the relationship between anhedonia and behavioral response to regrouping. During the first 8 and last 8 h of regrouping, agonistic behaviors initiated or received by the focal heifer were continuously recorded (WV-CW504SP, Panasonic, Osaka, Japan). Behaviors were collected according to Nogues et al. (2020). Briefly, these included displacements, replacements, avoidances, and fights (Table 1). Observers were not provided any information regarding the heifers' identification number nor were they aware of how the heifer had performed during the brush use test. Interobserver reliability scores were calculated using the intraclass correlation test in R (package “irr”) using a subset of videos watched by 2 observers; agreement between observers on all measures is shown in Table 1. The total number of negative interactions received was calculated by summing displacements, replacements, and threats. We also collected the time spent resting, using instantaneous scan sampling every 5 min. To assess feeding synchronicity, we counted the number of host heifers also feeding at each 5-min scan where the focal heifer was at the feedbunk. A synchronicity score was then calculated by averaging the number of host heifers that were feeding at the same time as the focal heifer; lower scores indicate that the focal heifer went feeding when the feed bunk was not occupied by many host heifers (avoid feeding peaks).

**Table 1:** Description of agonistic behaviors observed during regrouping and interobserver reliability scores

Behavior	Description	ICC^1^	(df) *F*	95% Confidence limit
Displacement	Push away another individual using head against another part of the body	0.99	(47,48) 138	0.975–0.992
Replacement	The heifer initiating the displacement also replaces the individual at the feeder or at the stall	0.80	(31,32) 9.05	0.633–0.897
Fight	Reciprocal head to head contact lasting more than 5 s	0.84	(7,8) 11.1	0.421–0.964
Active avoidance	Movement initiated presenting the forehead in direction of another heifer and resulting in the latter avoiding contact	0.80	(15,16) 11	0.597–0.938

1 Intraclass correlation coefficient.

We explored whether the individual variation in the change in brush use could be explained by agonistic behaviors received and behaviors expressed by heifers during the 8 h preceding the 2 brush tests. Two mixed linear models were built (using the PROC MIXED procedure in SAS) using the change in brush use 8 h and 56 h after regrouping as response variables. In both cases, the number of agonistic behaviors received, the time spent resting, and synchronization to feed in the 8 h preceding the brush tests (model 1: from 0 to 8 h; model 2: from 48 to 56 h) were added as fixed effects. To control for the variation originating from the host groups, host-group identity was included as a random effect in both models. Models were graphically checked for the normality of residuals and the presence of outliers.

We observed great variation in the frequency of agonistic interactions heifers received during the two 8-h periods of video watching (51 to 235 interactions on d 1; 12 to 84 interactions on d 3). Heifers also varied greatly in their behavioral response. Synchronization at the feeder ranged from 2.8 to 8.0 (on d 1) and from 3.3 to 8.5 (on d 3), and time spent resting ranged from 0 to 35.7% (on d 1) and from 8.3 to 45.8% (on d 3). However, the change (in percentage with respect to pre-regrouping values) in use of the brush 8 and 56 h after regrouping was not related to any of the other behaviors collected during the 8 h preceding both tests (all *P* > 0.05).

We expected that animals who (1) experienced more frequent agonistic interactions, (2) were less synchronized at the feeder, or (3) spent less time resting in the new freestall environment would show a greater reduction in brush use after regrouping. However, none of these hypotheses were supported by our results. Although these behaviors are often used as outcome measures in studies evaluating the effects of regrouping, no evidence to date links changes in these behaviors with how negatively regrouping is perceived by cattle.

The absence of a link between agonistic behaviors received and anhedonia is particularly surprising. There is abundant literature exploring negative affective states following social defeat in laboratory rodents (Rygula et al., 2005; Herzog et al., 2009; Papciak et al., 2013). However, some evidence indicates that persistent anhedonia may be triggered by social defeat only after chronic exposure (Yu et al., 2011) and not after a single episode (Razzoli et al., 2011). Arguably, heifers face many social defeat when regrouped (e.g., heifers experienced up to 235 displacements from the feeder over the first 8 h after regrouping in the current study), but this frequency may fail to capture the intensity of these negative interactions. Alternatively, our study may be underpowered, potentially explaining why we did not find a relationship. Future studies should consider increasing the sample size studied to explore why individuals vary in their affective response to regrouping.

The variation in mood change after regrouping may also be related to the perceived loss in social contact with familiar conspecifics. Previous work showed that calves form preferential social interactions (Raussi et al., 2010; Lecorps et al., 2019a), especially when they are raised in a stable group for a long time (Bolt et al., 2017), which was the case in our study. To the best of our knowledge, whether the negative effects associated with regrouping are due to the loss of a specific social companion have yet to be explored.

The change in physical environment may also be responsible for the change in mood. Some evidence suggests that being regrouped in a new environment is more detrimental than being regrouped in the home pen (Schirmann et al., 2011). In addition, cattle typically need some time to get used to freestalls (von Keyserlingk et al., 2011), and the most noticeable change in behavior is a decrease in resting time. Here, we expected that low resting time would negatively affect mood but this prediction was not supported by our results.

The individual variation may also be affected by differences in personality traits or dominance status. Recent evidence suggests that cattle vary in sociability (Gibbons et al., 2010; Lecorps et al., 2018) and aggressiveness (Gibbons et al., 2009), 2 traits that may affect their response to social confrontations that arise during regrouping. In addition, dominance status modulates the response to social confrontations (e.g., pigs: Otten et al., 1999), with results suggesting that higher loss in social status may be accompanied by higher negative affective states (pigs: Otten et al., 2002). We encourage future studies to explore whether personality traits and social status interact with the affective response to regrouping in cattle.

In conclusion, heifers displayed signs of anhedonia in the hours after regrouping. These results suggest that regrouping can lead to a negative mood state that is relatively short-lived. We found no evidence that anhedonia was affected by agonistic behaviors from the new group mates.
